# Author Correction: Tumour gene expression signature in primary melanoma predicts long-term outcomes

**DOI:** 10.1038/s41467-022-30365-w

**Published:** 2022-05-17

**Authors:** Manik Garg, Dominique-Laurent Couturier, Jérémie Nsengimana, Nuno A. Fonseca, Matthew Wongchenko, Yibing Yan, Martin Lauss, Göran B. Jönsson, Julia Newton-Bishop, Christine Parkinson, Mark R. Middleton, D. Timothy Bishop, Sarah McDonald, Nikki Stefanos, John Tadross, Ismael A. Vergara, Serigne Lo, Felicity Newell, James S. Wilmott, John F. Thompson, Georgina V. Long, Richard A. Scolyer, Pippa Corrie, David J. Adams, Alvis Brazma, Roy Rabbie

**Affiliations:** 1 European Molecular Biology Laboratory, European Bioinformatics Institute (EMBL-EBI), Hinxton, Cambridgeshire UK; 2 Cancer Research UK Cambridge Institute, University of Cambridge, Li Ka Shing Centre, Robinson Way, Cambridge, UK; 3 University of Leeds School of Medicine, Leeds, United Kingdom; 4 Biostatistics Research Group, Population Health Sciences Institute, Faculty of Medical Sciences, Newcastle University, Newcastle upon Tyne, UK; 5 CIBIO/InBIO-Centro de Investigação em Biodiversidade e Recursos Genéticos, Universidade do Porto, Rua Padre Armando Quintas, 4485-601 Vairão, Portugal; 6 Oncology Biomarker Development, Genentech Inc., 1 DNA Way, South San Francisco, CA 94080 USA; 7 Lund University Cancer Center, Lund University, Lund, Sweden; 8 Cambridge Cancer Centre, Cambridge University Hospitals NHS Foundation Trust, Cambridge, UK; 9 Oxford NIHR Biomedical Research Centre and Department of Oncology, University of Oxford, Oxford, UK; 10 Department of Pathology, Cambridge University Hospitals NHS Foundation Trust, Cambridge, UK; 11 Melanoma Institute Australia, The University of Sydney, North Sydney, NSW Australia; 12 Faculty of Medicine and Health, The University of Sydney, Sydney, NSW Australia; 13 Institute for Research and Medical Consultations (IRMC), Imam Abdulrahman Bin Faisal University, Dammam, Saudi Arabia; 14 QIMR Berghofer Medical Research Institute, Brisbane, QLD Australia; 15 Discipline of Surgery, Faculty of Medicine and Health, The University of Sydney, Sydney, NSW Australia; 16 Royal North Shore and Mater Hospitals, Sydney, Australia; 17 Tissue Pathology and Diagnostic Oncology, Royal Prince Alfred Hospital and New South Wales Health Pathology, Sydney, NSW Australia; 18 Experimental Cancer Genetics, The Wellcome Sanger Institute, Hinxton, Cambridgeshire UK

**Keywords:** Cancer genomics, Melanoma, Tumour biomarkers

Correction to: *Nature Communications* 10.1038/s41467-021-21207-2, published online 18 February 2021.

The original version of this article used a registered trademark name to indicate the 27 genes that comprise the ‘DecisionDx-Melanoma’ test; which was incorrect References to the gene signature derived from these genes have been amended and are now referred to as the ‘Gerami_27’ gene signature.

In particular:

In the fourth paragraph of the Results section “Signature added incremental prognostic value when combined with conventional clinical staging” the sentence “The published signature from Gerami et al.^12^ (Decision-Dx MelanomaTM; *n* = 27 genes) was not associated with OS in multivariate models in the AVAST-M primary melanoma dataset” has been replaced with “The published signature from Gerami et al.^12^ (Gerami_27; *n* = 27 genes) was not associated with OS in multivariate models in the AVAST-M primary melanoma dataset”.

In the third paragraph of the Results section “Cam_121 predicts metastasis better than both clinical covariates and published prognostic signatures” the sentence “In order to test the performance of the published prognostic signatures from Gerami et al. (Decision-Dx MelanomaTM; *n* = 27 genes) and Thakur et al.” has been replaced with “In order to test the performance of the published prognostic signatures from Gerami et al. (Gerami_27; *n* = 27 genes) and Thakur et al.”. In the same section in the last line “Cam_121 vs Decision-Dx Melanoma” has been replaced with “Cam_121 vs Gerami_27”.

In the first paragraph of the Discussion, the sentence “The 31-GEP assay (Decision-Dx MelanomaTM, Castle Biosciences) has been developed in an attempt to address this clinical dilemma” has been replaced with “The 31-GEP assay (Gerami_27) has been developed in an attempt to address this clinical dilemma”.

In the first paragraph of Methods section “Model development and selection”, the sentence “We also compared this to the predictive power of two published prognostic signatures (LMC_150 and Decision-Dx Melanoma” has been replaced with “We also compared this to the predictive power of two published prognostic signatures (LMC_150 and Gerami_27’ in an independent analysis”.

In Fig. 3e, Decision-Dx’ has been replaced with: ‘Gerami_27’ and in the associated legend: the sentence “Decision-Dx Melanoma: Decision-Dx MelanomaTM, LMC_150: Leeds Melanoma Cohort 150 gene signature” has been replaced with “Gerami_27, LMC_150: Leeds Melanoma Cohort 150 gene signature”.

The original reference 12 “Gerami, P. et al. Gene expression profiling for molecular staging of cutaneous melanoma in patients undergoing sentinel lymph node biopsy. J Am Acad Dermatol 72, 780-5.e3 (2015)” has been replaced with “Gerami, P. et al. Development of a prognostic genetic signature to predict the metastatic risk associated with cutaneous melanoma. Clin Cancer Res 21, 175-183 (2015).

In Supplementary Fig. 6a the *x*-axis label ‘Decision-Dx’ has been replaced with ‘Gerami_27’.

The title of Supplementary Fig. 6a has been updated to read ‘Forest plot of univariate and multivariate survival analysis for the two previously published signatures (Gerami_27 and LMC_150). In the legend to Supplementary Figure 6a the sentence that previously read ‘Forest plot indicating the hazard ratio (HR) estimates (dots at the centre of error bars), corresponding to 95% confidence intervals of the HR estimates (horizontal error bars) and two-sided Wald t-test p-values related to the signature parameter when considering the signature definitions of a) Gerami and b) LMC_150 when predicting overall survival (green) and progression free survival (orange) by means of Cox proportional hazard models when controlling for different (sets of) clinical variables (y-axes)’ has been updated to read ‘Forest plot indicating the hazard ratio (HR) estimates (dots at the centre of error bars), corresponding to 95% confidence intervals of the HR estimates (horizontal error bars) and two-sided Wald t-test p-values related to the signature parameter when considering the signature definitions of a) Gerami_27 and b) LMC_150 when predicting overall survival (green) and progression free survival (orange) by means of Cox proportional hazard models when controlling for different (sets of) clinical variables (y-axes).

In row 4 of Supplementary Tables 2c ‘Decision-Dx’ has been replaced with ‘Gerami_27’.

In addition, the original Supplementary Fig. 3a and Supplementary Fig. 15 (top) contained a typographical error and ‘no distant recurrence, 89’ has been replaced with ‘no distant recurrence, 105’.

The text and the main figures have been corrected in both the PDF and HTML versions of the Article. The HTML has been updated to include a corrected version of the Supplementary Information.

## Supplementary information

Updated Supplementary Data

