# Supplementary material for: Tumour gene expression signature in primary melanoma predicts long-term outcomes
Source: Nat Commun. 2021 Feb 18;12:1137. doi: 10.1038/s41467-021-21207-2 (PMC7893180; doi:10.1038/s41467-021-21207-2)
Supplement: Supplementary file 1 — Updated Supplementary Information [file 41467_2021_21207_MOESM1_ESM.pdf]

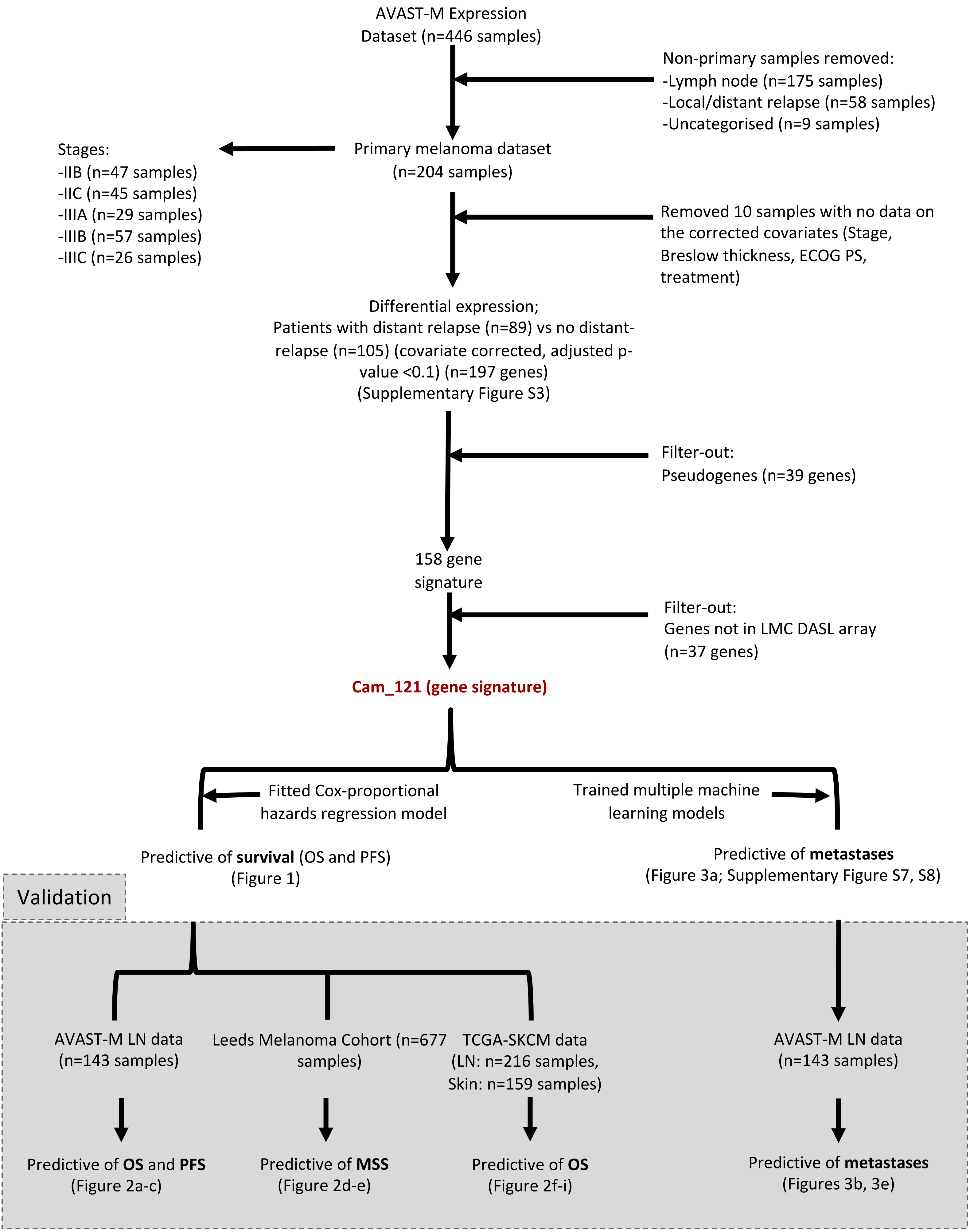

Supplementary Figure S1. Flow chart of the analysis. The figures corresponding to each specified analysis are indicated. OS: Overall survival; PFS: progression-free survival; MSS: melanoma-specific survival; LN: lymph node. ECOG: Eastern Cooperative Oncology Group Performance Status.

a

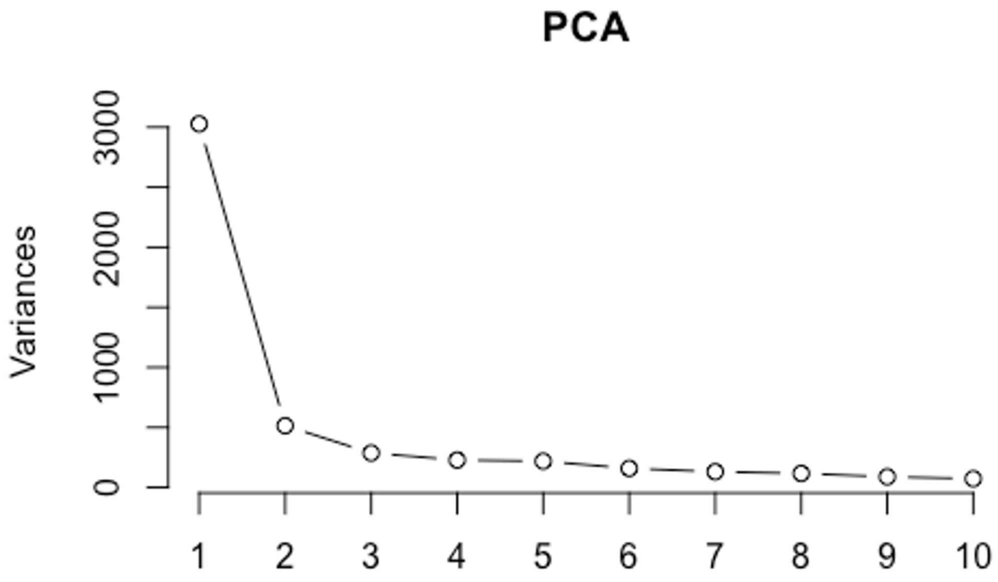

b

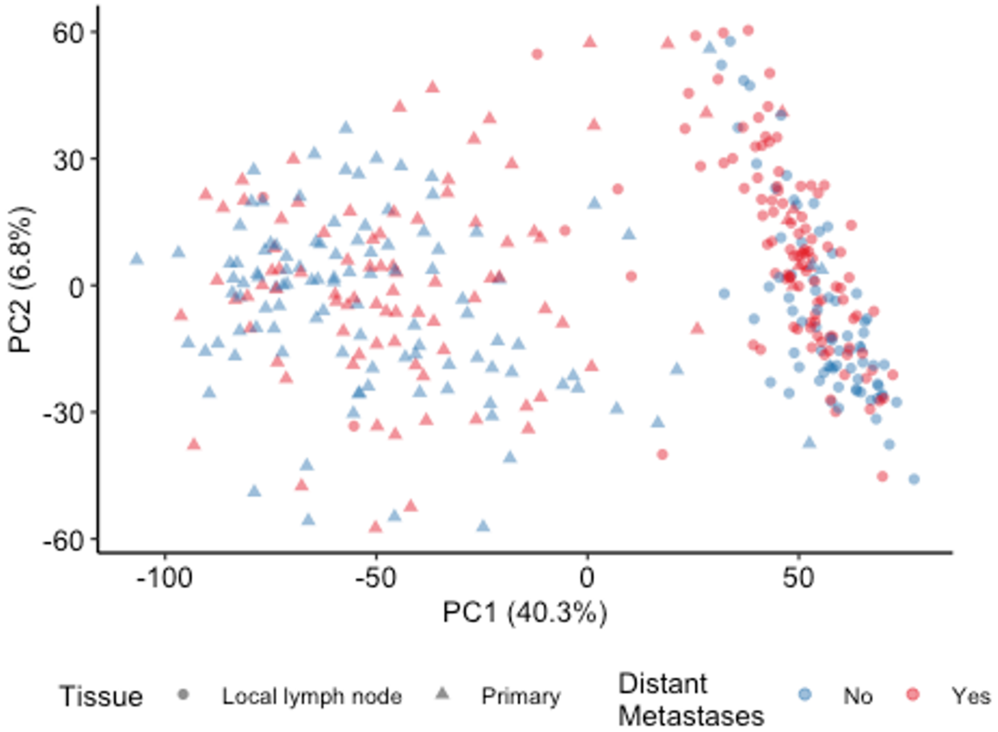

Supplementary Figure S2. PCA of the 1000 most variable genes, showing that samples separated by their tissue site of origin. a) Scree-plot showing the variance of the data (y-axis) explained by each of the 10 first principal components (x-axis). **b)** Biplot of the scores of the observations on the two first dimensions of a PCA analysis on the 1000 most variable genes. The samples are colour-coded according to their relapse status (yes in red, no in blue) and shaped according to their tissue of origin (primary melanoma samples in triangles, lymph nodes in circles). The x-axis of the biplot, accounting 40.3% of the total variance, shows that samples are clustered primarily based on the tissue site of origin. Source data are provided as a Source Data file.

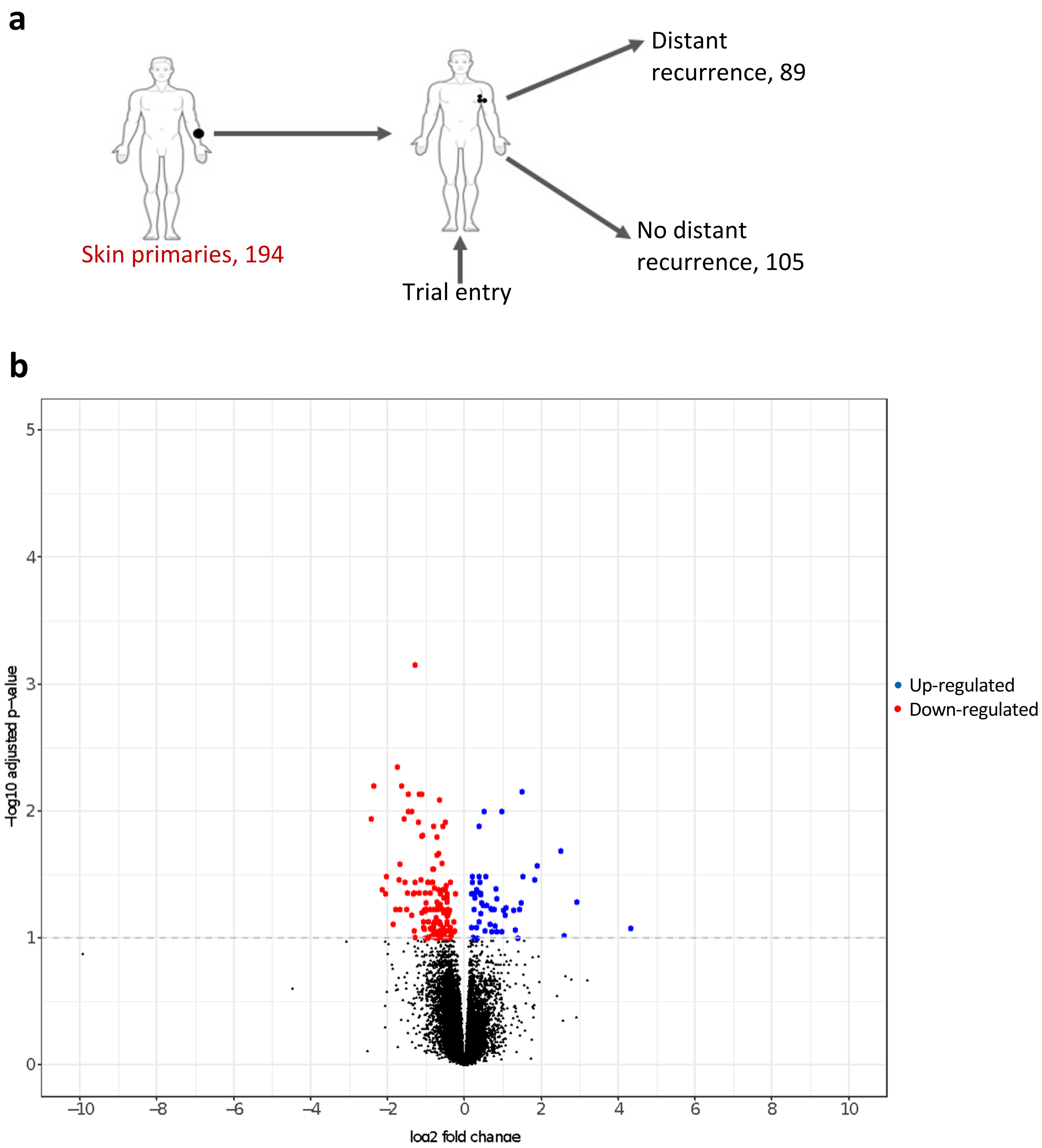

Supplementary Figure S3. Covariate-corrected differential expression analysis for primary melanoma samples (comparing metastases vs non-metastases). a) Schematic flowchart indicating the number of samples considered in the differential expression analysis. **b)** Volcano plot showing, for each gene, the -log 10 FDR corrected p-value (y-axis) and the corresponding log-fold change estimate within the differential expression analysis (x-axis). The (predominant) downregulated (144/197, 73.1%) and upregulated (53/197, 26.9%) DEGs are respectively coloured in red and blue for genes with a p-value < 0.1 after Benjamini and Hochberg multiplicity correction (this p-value cut-off is represented by the dashed horizontal grey line). Source data are provided as a Source Data file.

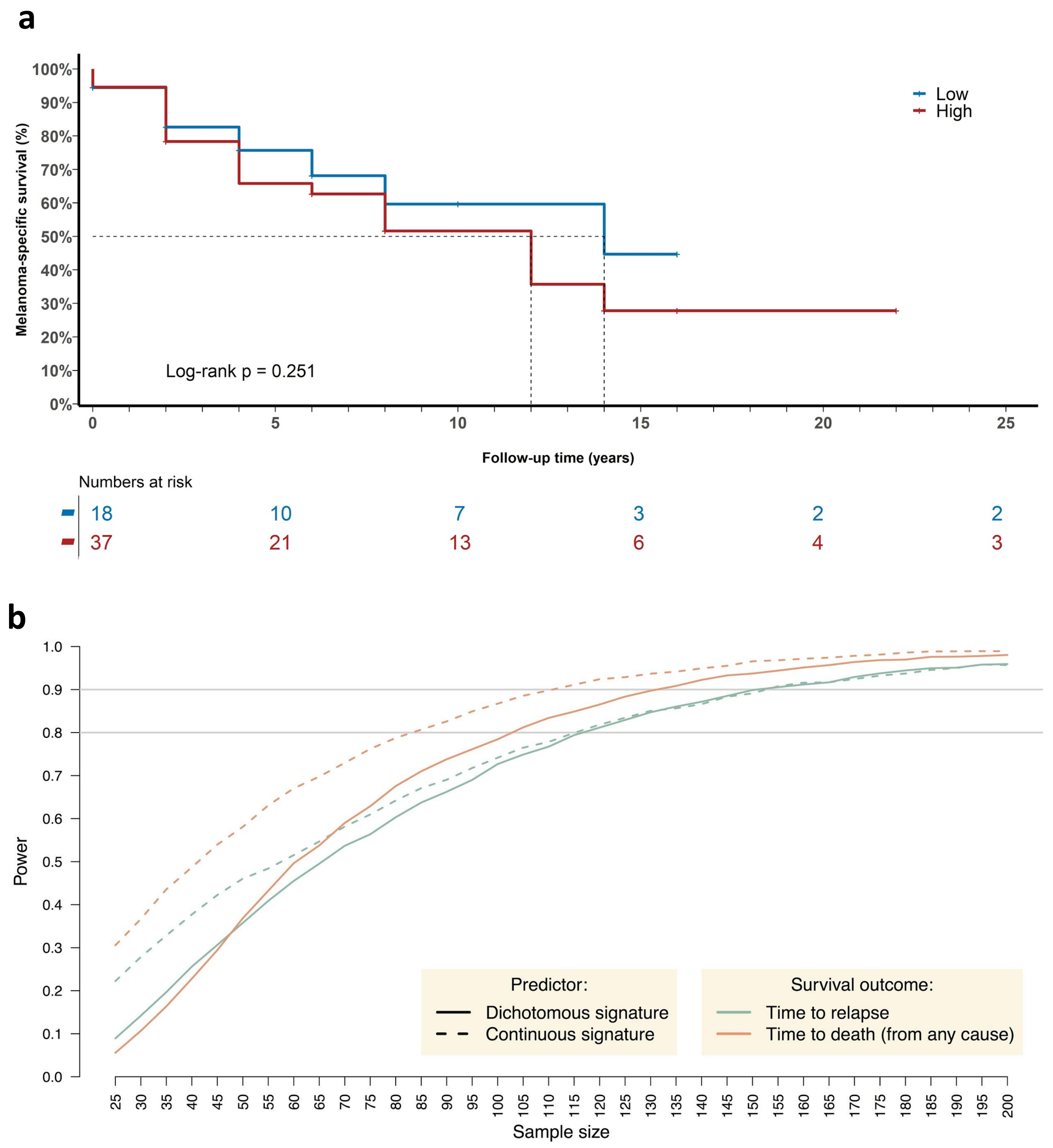

Supplementary Figure S4: Validation of Cam_121 within a third independently acquired external dataset (Australia Melanoma Genome Project, n=55). a) Kaplan-Meier curve comparing the melanoma specific survival probabilities (y-axis) of groups with high and low "Cam_121" (quantile 0.33 split) as a function of follow-up time in years (x-axis). Note that this dataset contains samples (n=55) from a variety of tissue sites (including primary tumours, regional lymph nodes, distant metastases, in-transit metastases and others) The p-value of a two-sided logrank test comparing the survival distributions of both groups is indicated. **b)** Simulation-based power analysis based on the AVAST-M study considering R=2500 Monte Carlo samples. The plot shows the power (y-axis) as a function of the sample size (x-axis) when modelling two survival outcomes, the time to relapse (green lines) and time to death from any cause (red lines), as a function of our signature, both considered as a dichotomous (solid line) and continuous (dashed line) predictor. A sample size greater than n=115 would be required to obtain a power greater than 80% in all cases.

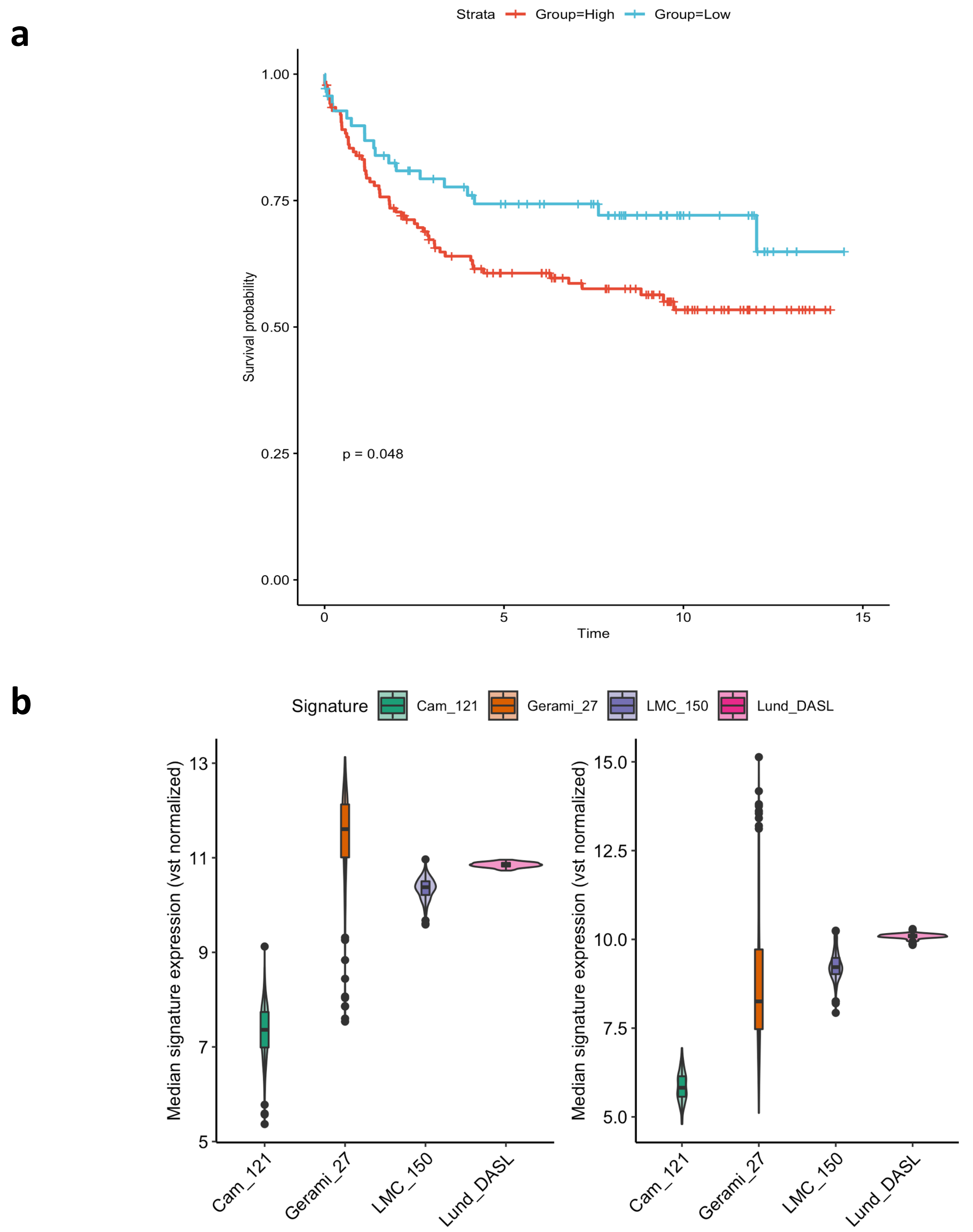

Supplementary Figure S5. Partial validation of Cam_121 within a fourth independently acquired external dataset (Lund Melanoma Cohort, n=223). a) Kaplan-Meier plot comparing the progression free survival probabilities (y-axis) of groups with high and low "Cam_121" (quantile 0.33 split) as a function of time in years (x-axis) within the Lund Melanoma Cohort (n=223) and p-value of a two-sided logrank test comparing the survival distributions. Note that only 24 genes out of 121 were identified within the Lund dataset. The Wald t-test p-values corresponding to the signature "Cam_121" is indicated. **b)** Violin plots showing the median gene expression values (normalized using variance stabilizing transformation (vst)) of the genes (y-axes) in each signature (x-axes). Note that for the signature "Lund_DASL" (pink), all the 7739 genes present in the Lund Melanoma Cohort were used for calculating the median expression value in each dataset. Left panel: median expression within the AVAST-M primary melanoma dataset (n=194), right panel: median expression within TCGA-SKCM Skin dataset (n=159). The median (vst normalized) expression values for each signature were plotted using the *geom_violin* and *geom_boxplot* function of the R-package ggplot2. The center of the box-plot denotes the median value; the bounds of the box denote the 25th percentile and the 75th percentile values; the dots denote the outliers lying ± 1.5 times beyond the interquartile range from the bounds of the box (shown by the minima and maxima of the box-plot).

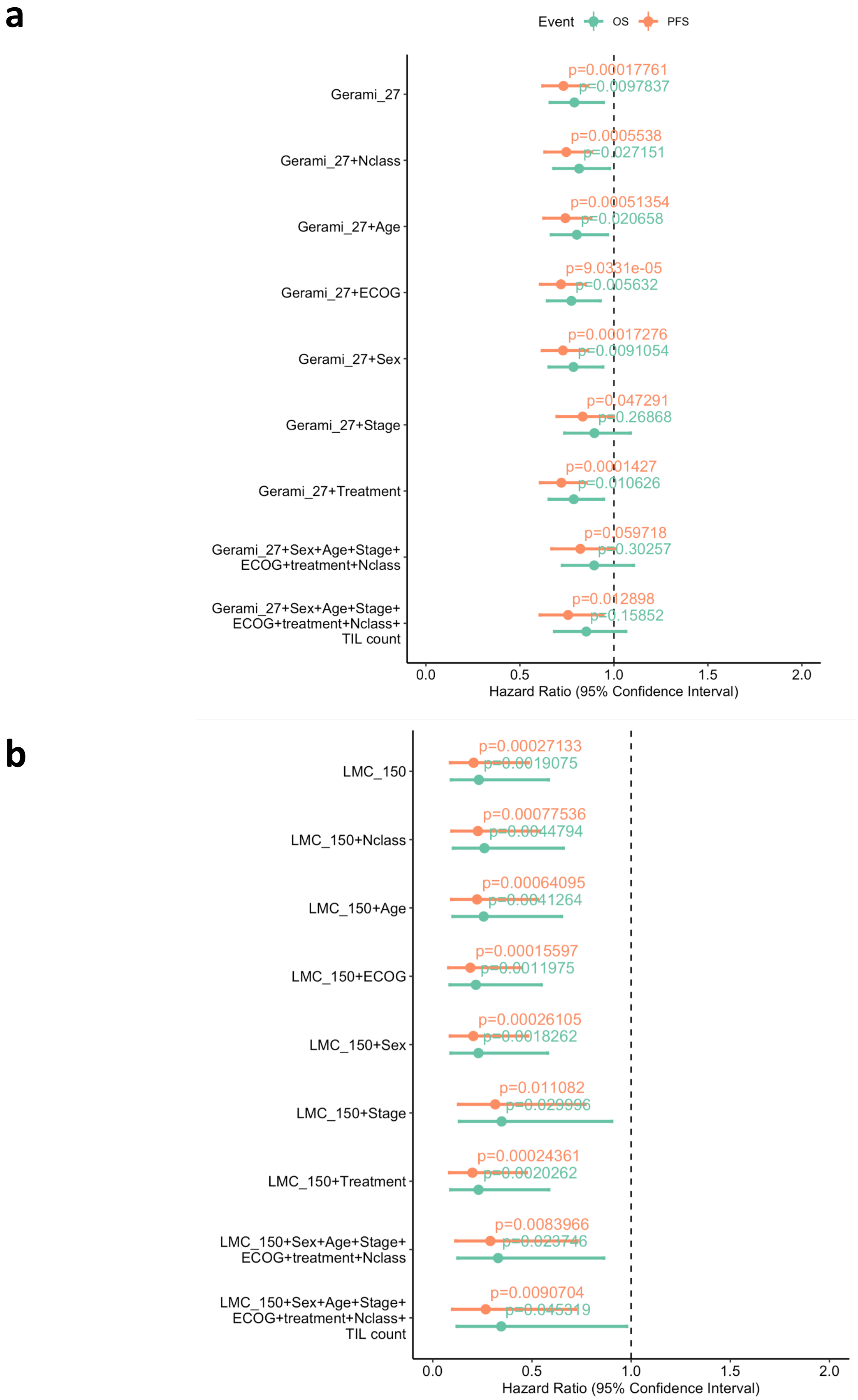

Supplementary Figure S6. Forest plot of univariate and multivariate survival analysis for the two previously published signatures (Gerami_27 and LMC_150). Forest plot indicating the hazard ratio (HR) estimates (dots at the centre of error bars), corresponding to 95% confidence intervals of the HR estimates (horizontal error bars) and two-sided Wald t-test p-values related to the signature parameter when considering the signature definitions of **a)** Gerami_27 and **b**) LMC_150 when predicting overall survival (green) and progression free survival (orange) by means of Cox proportional hazard models when controlling for different (sets of) clinical variables (y-axes). No multiplicity correction were used. ECOG: Eastern Cooperative Oncology Group Performance Status. TIL count; Tumour-infiltrating lymphocyte count.

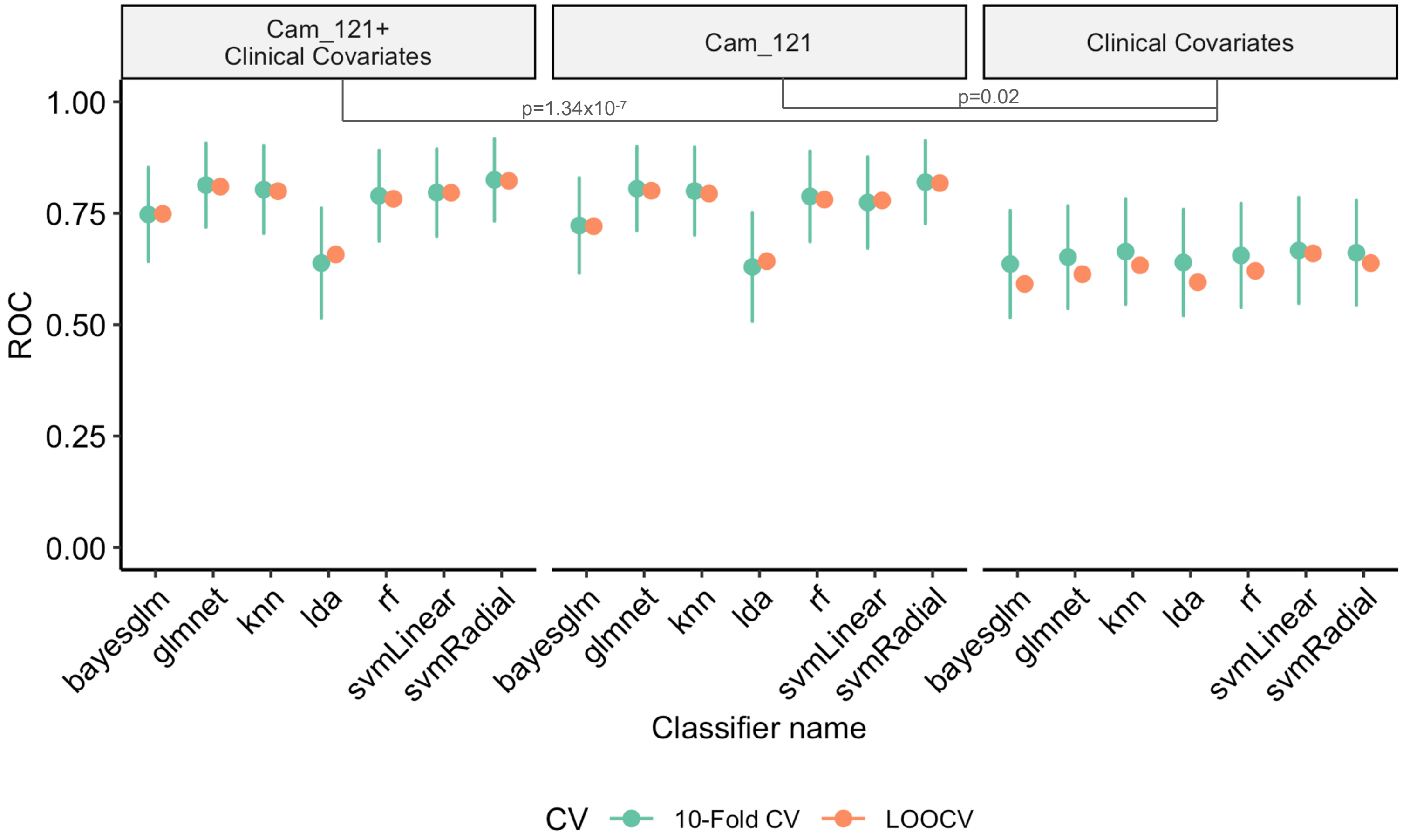

Supplementary Figure S7. Comparison of 10-Fold and Leave-one out cross validation within the machine learning model (training). Plot showing, for each set of predictors (panels) and 7 different classification models (x-axis), the mean ± sd AUROC obtained from 10-Fold cross validation in green (repeats = 1000) and the AUROC estimates from leave-one-out cross-validation in orange. The final classification remained consistent across both cross-validation approaches. The p-values of statistical comparisons using one-sided two-sample Welch t-tests are indicated (see also supplementary table S2a). See methods section 10 for details about the classification algorithms. Source data are provided as a Source Data file.

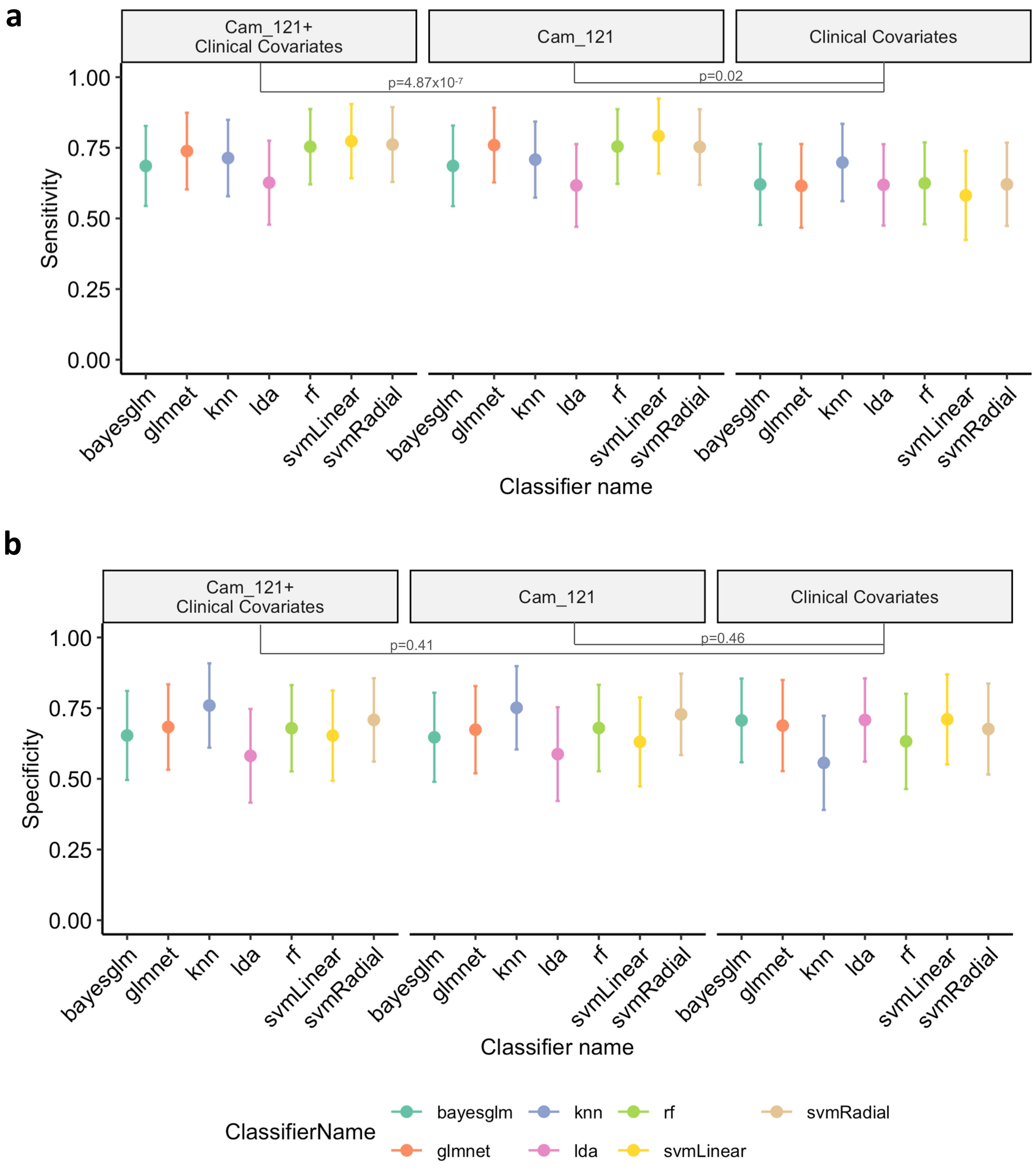

Supplementary Figure S8. Machine learning results across multiple classifiers, showing that Cam_121 outperforms clinical covariates using 10-Fold CV (repeats = 1000). Plots showing, for each set of predictors (columns), the mean ± sd scores of **a)** sensitivity **b)** and specificity (y-axes) of the 7 selected classifiers (x-axes). The p-values of statistical comparisons using one-sided two-sample Welch t-tests are indicated (see also supplementary table S2a). See methods section 10 for details about the classification algorithms. Source data are provided as a Source Data file.

a

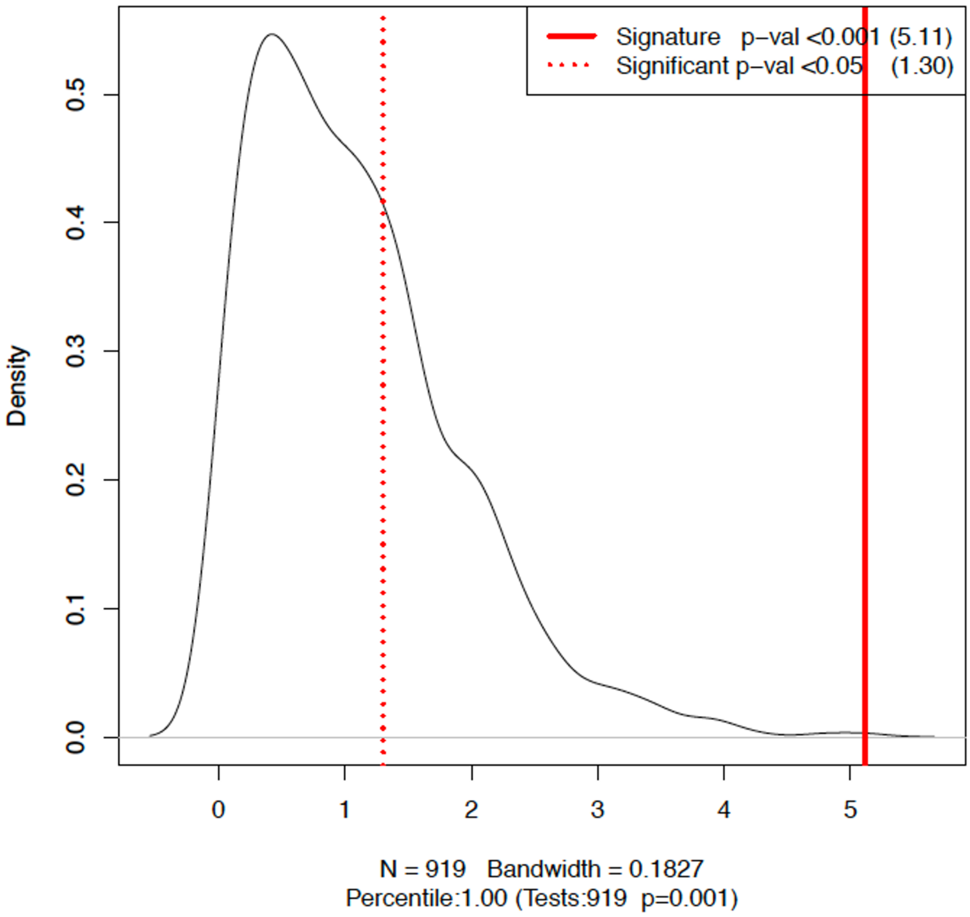

b

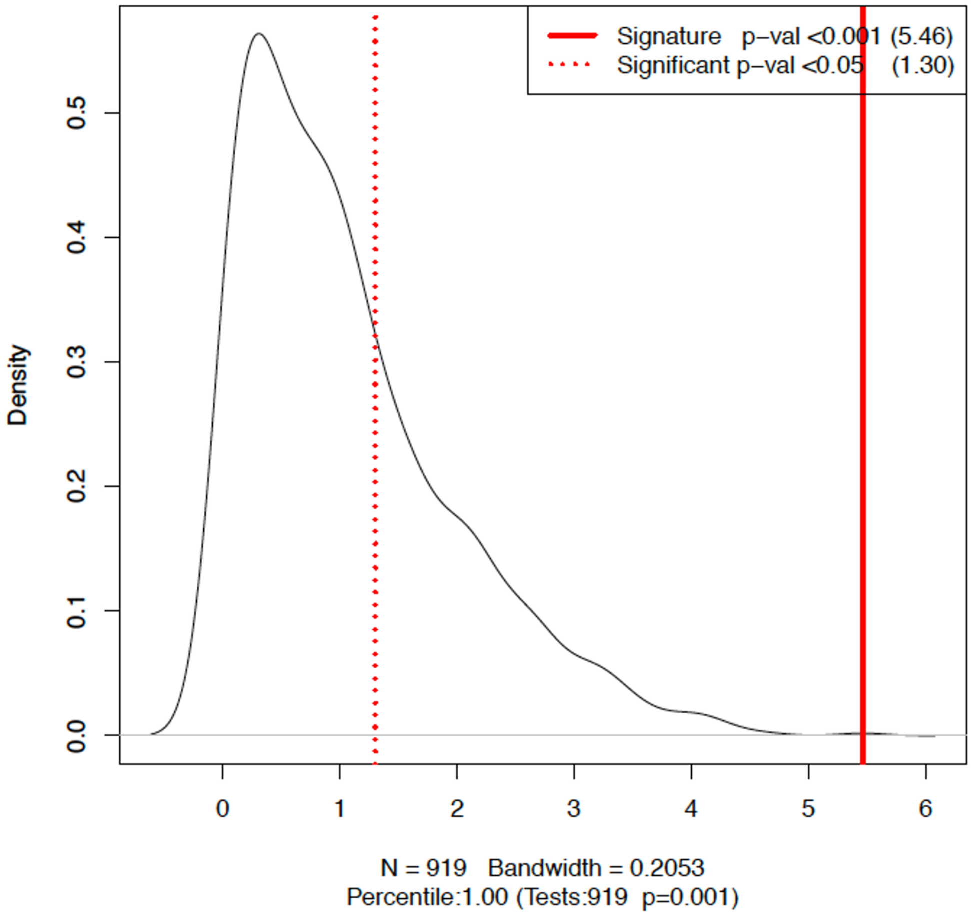

Supplementary Figure S9. The Cam_121 signature significantly outperforms 1000 sets of 121 randomly selected protein-coding genes. Plots showing the densities of the p-values corresponding to the Cam_121 gene signature when considering random signatures (smoothed densities) in terms of **a)** OS and **b)** PFS in multivariate Cox regression models. The one-sided p-values obtained with the Cam_121 signature appear as red vertical lines. The p-values are defined as the fraction of scores of random signatures which are greater than those observed using the (real) Cam_121 gene signature. The dotted red vertical lines correspond to -log10(0.05), i.e., to the 5% cutoff meaning that only 5% of the p-values (on the -log10 scale) corresponding to random signatures would be beyond this point if random signatures had no predictive effect. In **a)** and **b)** No multiplicity corrections were used to define the p-values indicated in the legend.

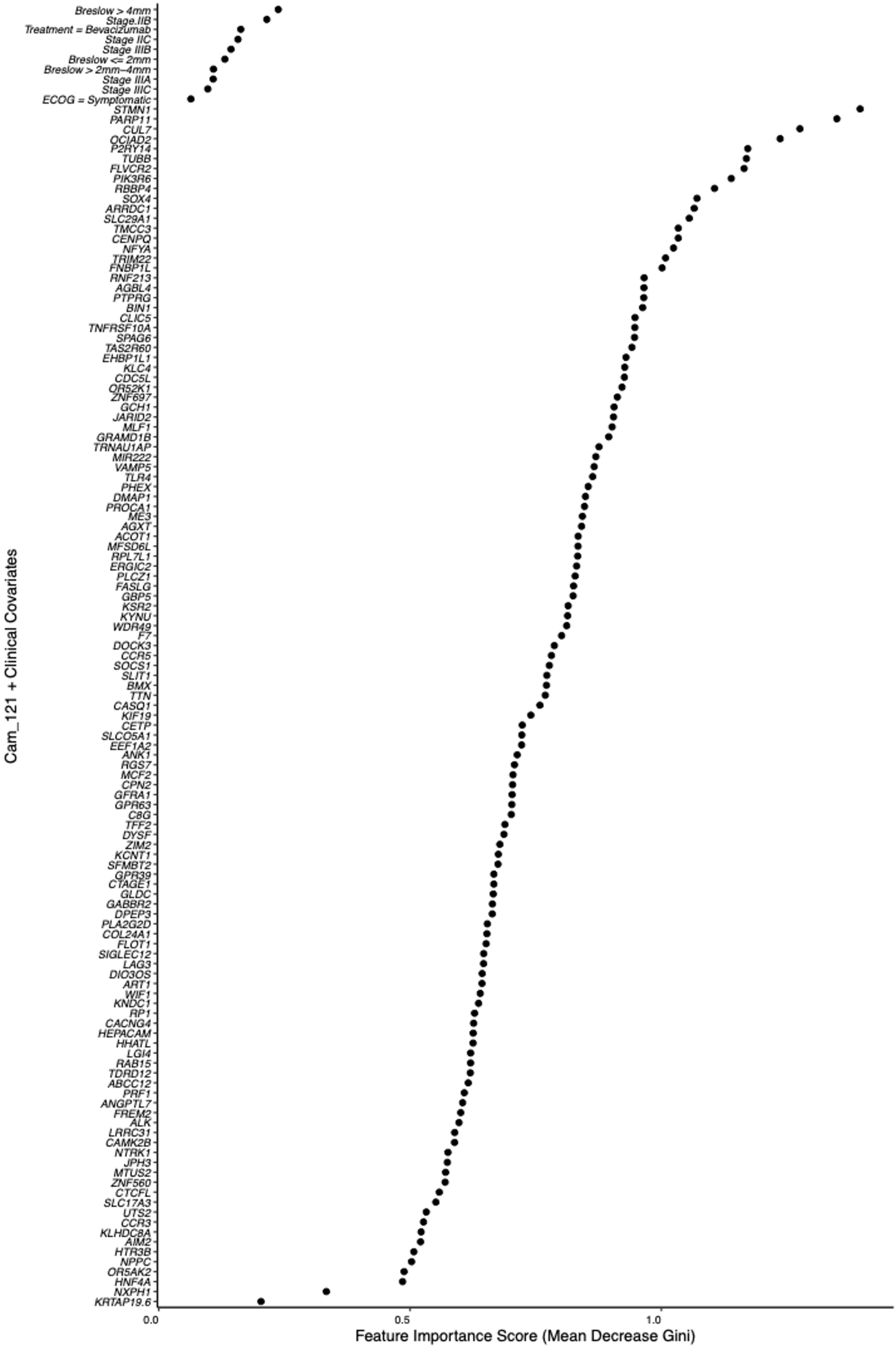

Supplementary Figure S10. Feature importance score (FIC) for each gene in Cam_121 showing similar scores across all genes within the random forest machine learning model suggesting that, rather than the dominance of any single gene, it is a composite analysis of all genes that contribute to the signatures' prognostic power. Genes are ranked in decreasing FIC order. The clinical covariates are indicated on the top for comparison. Source data are provided as a Source Data file.

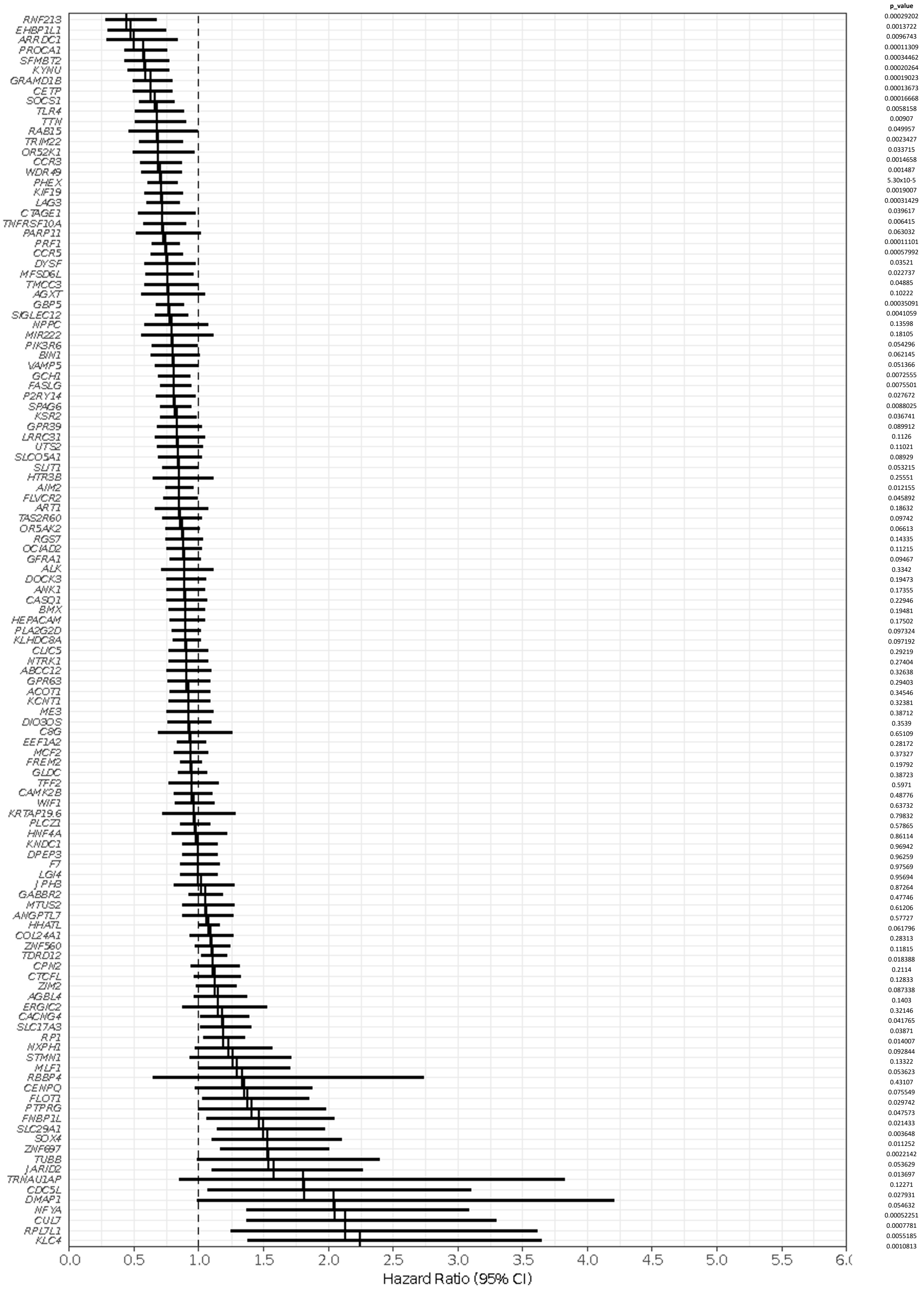

Supplementary Figure S11. Multivariate survival analysis (OS) for each Cam_121 signature gene. Forest plot indicating the hazard ratio (HR) estimates (vertical lines at the centre of error bars) and horizontal error bars corresponding to 95% confidence intervals of the HR estimates for each Cam_121 gene ranked in increasing order of HR. The corresponding two-sided Wald t-test p-values (before FDR correction) are indicated on the right. No single gene proved significant after correction for multiple testing (FDR corrected p-value<0.05). This therefore suggests that it is a composite analysis of all genes that contribute to the signatures' prognostic power rather than the dominance of any single gene. No multiplicity correction were used.

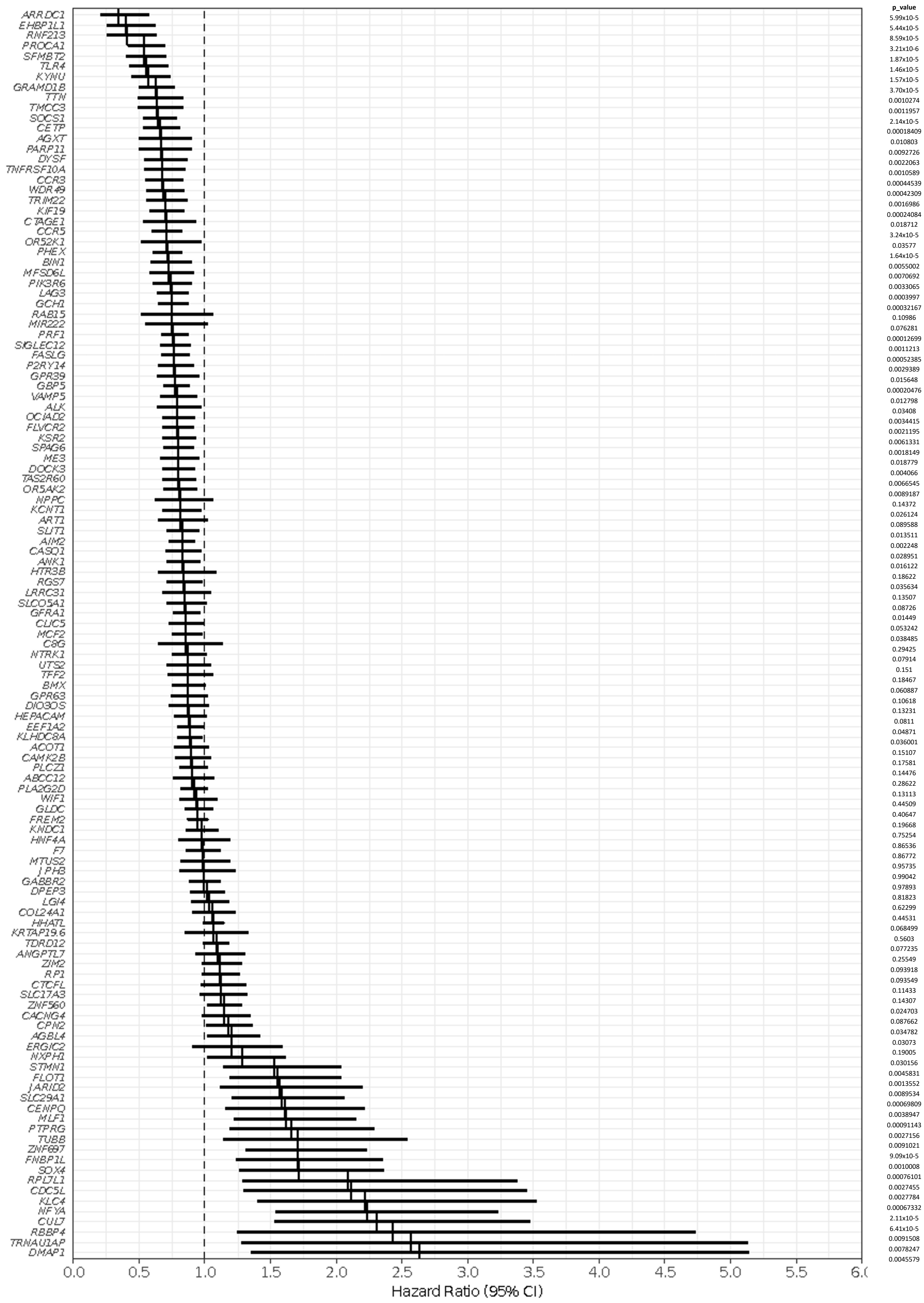

Supplementary Figure S12. Multivariate survival analysis (PFS) for each Cam_121 signature gene. Forest plot indicating the hazard ratio (HR) estimates (verrical lines at the centre of error bars) and horizontal error bars corresponding to 95% confidence intervals of the HR estimates for each Cam_121 gene ranked in increasing order of HR. The corresponding two-sided Wald t-test p-values (before FDR correction) are indicated on the right. No single gene proved significant after correction for multiple testing (FDR corrected p-value<0.05). This therefore suggests that it is a composite analysis of all genes that contribute to the signatures' prognostic power rather than the dominance of any single gene. No multiplicity correction were used.

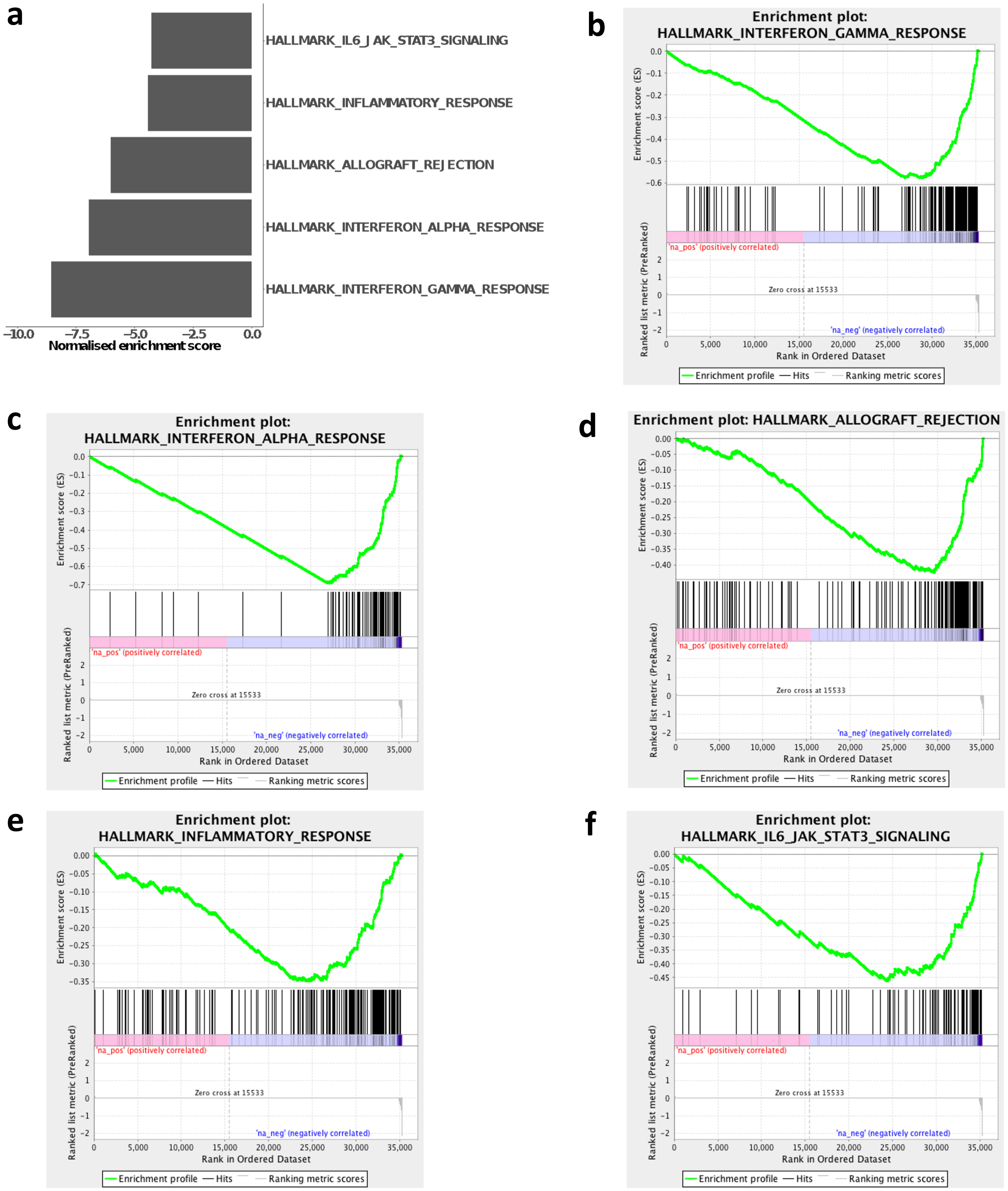

Supplementary Figure S13. Results of gene set enrichment analysis showing downregulation of key immune-related pathways in primary melanoma samples destined for metastases. a) Barchart of the top five most significant down-regulated Hallmark gene sets ($p<0.01$ for all gene sets). **b-f)** The corresponding enrichment plots from (a). The top portion of the plot shows the running enrichment score for the gene set as the analysis walks down the ranked list. The middle portion of the plot shows where the members of the gene set appear in the ranked list of genes and the bottom portion of the plot shows the value of the ranking metric as one moves down the list of ranked genes. Source data are provided as a Source Data file.

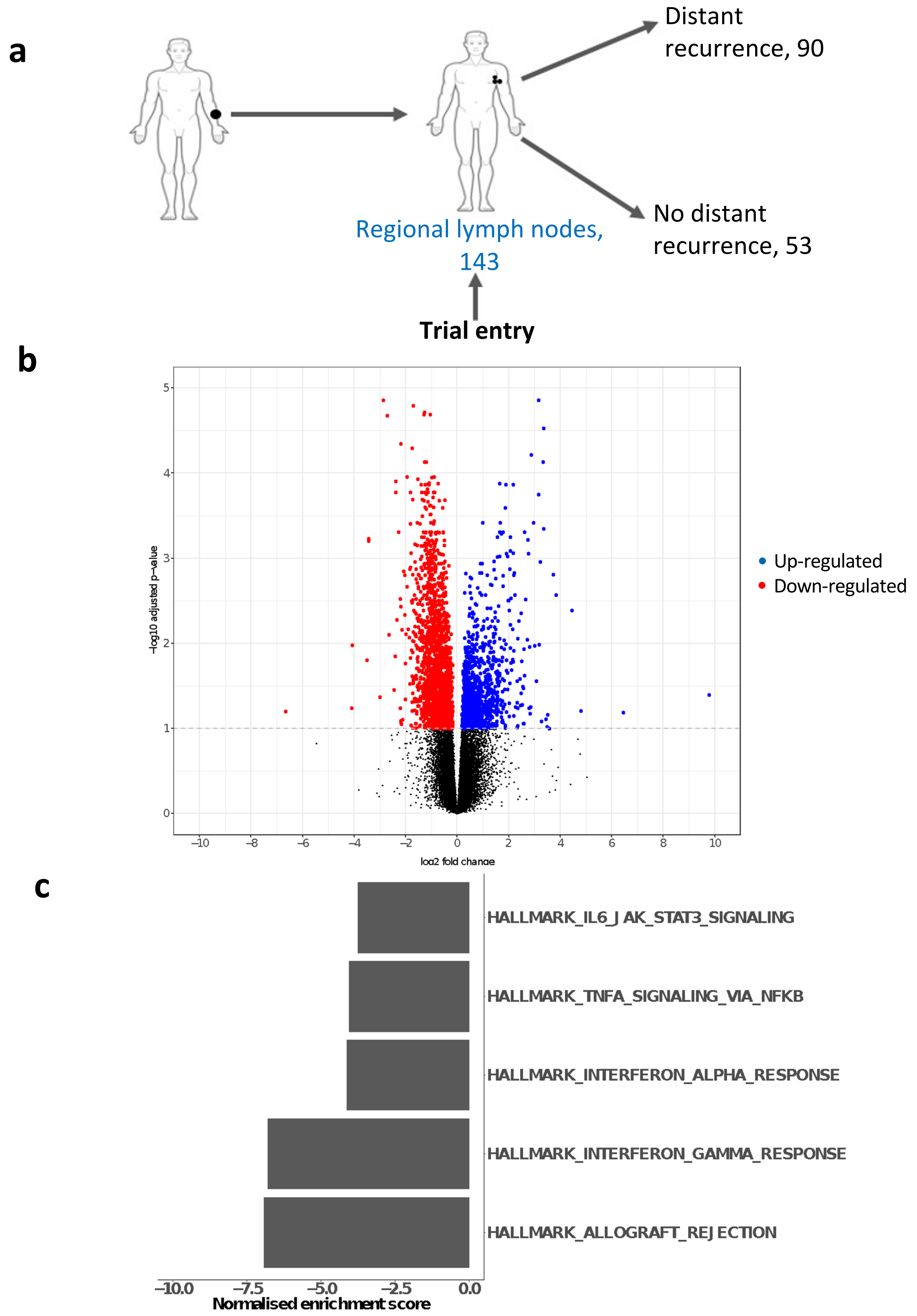

Supplementary Figure S14. Results of differential expression and gene-set enrichment analysis comparing metastases vs non-metastases in lymph node samples (n=143), uncovering the same results as those obtained from primary melanoma samples. a) A schematic of the number of samples in the covariate corrected differential expression analysis. **b)** Volcano plot showing, for each gene, the -log 10 FDR corrected p-value (y-axis, FDR<0.1) and the corresponding log-fold change estimate within the differential expression analysis (x-axis). The (predominant) downregulated (1967/3022 (65.1%)) and up upregulated DEGs (1055/3022 (34.9%)) are respectively colour-coded in red and blue for genes showing a p-value < 0.1 after Benjamini and Hochberg multiplicity correction. **c)** Barplot showing downregulation of the same top five immune-related pathways as those identified in primary melanoma sample analyses (FDR corrected p-value<0.01 for all gene sets) (Supplementary Figure S13A). Source data are provided as a Source Data file.

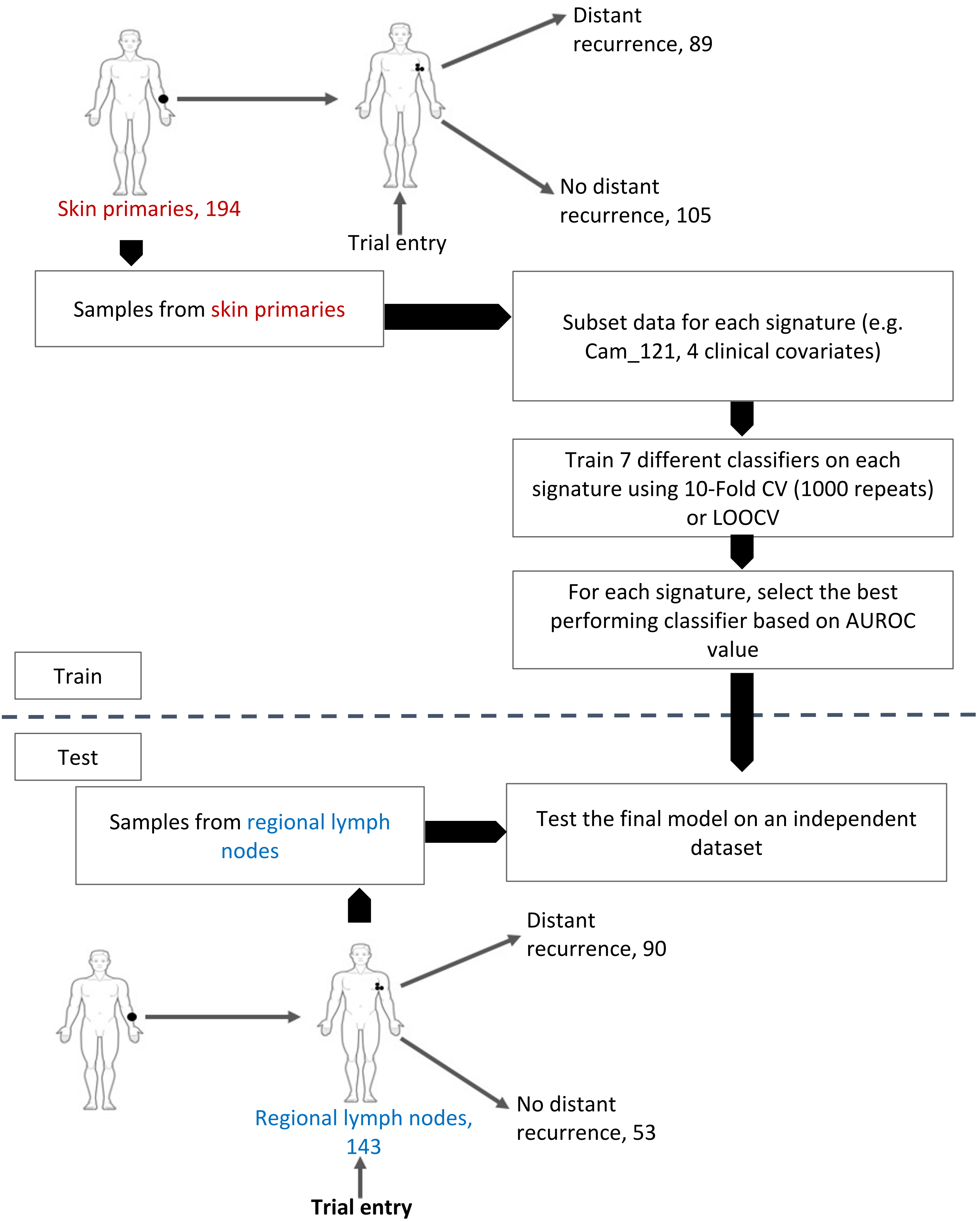

Supplementary Figure S15. Schematic outline of the machine learning analysis. The AVAST-M primary melanoma dataset was used for training the machine learning and the AVAST-M lymph node dataset was used for testing the final model. Three different sets of features were considered for comparison: (i) considering the Cam_121 gene expression values only as features, (ii) one-hot encoded clinical covariates only as features, (iii) Cam_121 gene expression values as well as one-hot encoded clinical covariates as features. For each set of features, 7 different classifiers were trained using 10-Fold cross-validation (CV repeats = 1000) or leave-one-out cross validation (LOOCV) and the classifier giving the highest mean area under the ROC curve (AUROC) value was selected as the final model (see methods section 10).

Supplementary Table S1a: Analyses of the clinical covariates associated with metastases: relapsed (n=590) vs non-relapsed (n=753). Corrected for the length of follow-up and treatment. The likelihood ratio test p-values were calculated and corrected for multiple testing using Holm-Bonferroni method (see Methods section 8).

Clinical Covariate	p-value	Adjusted p-value
Stage	1.01x10-25	1.11x10-24
Nclass	1.07x10-21	1.07x10-20
ECOG	0.0036	0.03
Treatment	0.045	0.36
Age	0.076	0.53
BRAF	0.1	0.6
Sex	0.56	1
Site	0.79	1
Breslow thickness	0.22	1
Ulceration	0.68	1
NRAS	0.21	1

Supplementary Table S1b: Analyses of the clinical covariates associated with overall survival. The p-values from the partial-likelihood-ratio tests performed on the coxph model objects are reported.

Clinical Covariate	Pr(>|Chi|)
EventMet	4.90x10-157
Sex	4.61x10-3
Age	3.20x10-3
I(Age^2)	3.04x10-1
as.numeric(Nclass)	1.42x10-2
Stage	8.78x10-2
Site	7.70x10-1
Breslow thickness	6.43x10-1
Ulceration	8.40x10-1
Treatment	4.80x10-1
ECOG	2.14x10-2

Supplementary Table S1c: Analyses of the clinical covariates associated with progression-free survival. The p-values from the partial-likelihood-ratio tests performed on the coxph model objects are reported.

<table>
<tr><th>Clinical Covariate</th><th>Pr(>|Chi|)</th></tr>
<tr><td>EventMet</td><td>1.73x10-278</td></tr>
<tr><td>Sex</td><td>1.55x10-1</td></tr>
<tr><td>Age</td><td>1.03x10-1</td></tr>
<tr><td>I(Age^2)</td><td>6.61x10-1</td></tr>
<tr><td>as.numeric(Nclass)</td><td>5.81x10-3</td></tr>
<tr><td>Stage</td><td>1.76x10-3</td></tr>
<tr><td>Site</td><td>4.28x10-1</td></tr>
<tr><td>Breslow thickness</td><td>3.72x10-2</td></tr>
<tr><td>Ulceration</td><td>4.51x10-1</td></tr>
<tr><td>Treatment</td><td>8.68x10-1</td></tr>
<tr><td>ECOG</td><td>7.22x10-1</td></tr>
</table>

Supplementary Table S2a: Statistical analysis of mean classifier results shown in Figure 3a and Supplementary Figure S8. The mean performances of 7 classifiers for signatures were compared in a pairwise manner using one-sided two-sample Welch t-tests implemented in R by the function t.test() with parameters (var.equal = FALSE, paired = FALSE, alternative = "greater"). No multiplicity correction were used.

	ROC (p-value)		Sensitivity (p-value)		Specificity (p-value)	
Signature > Clinical covariates	p-value	z-statistic	p-value	z-statistic	p-value	z-statistic
Cam_121 + Clinical Covariates > Clinical Covariates	1.34E-07	8.05E+00	4.87E-07	6.5796	0.4079	0.2353
Cam_121 > Clinical Covariates	0.0023	4.2749	0.0018	3.8075	0.4634	0.0937

Supplementary Table S2b: Statistical analysis of ROC curves shown in Figure 3b. The ROC curves were compared using the roc.test() function in R with parameters (method="delong", paired=FALSE, alternative = "greater"). The one-sided DeLong's p-values and the test statistic for each comparison are reported. No multiplicity correction were used.

Signature > Clinical covariates	DeLong's p-value	DeLong's Z-statistic
Cam_121 + Clinical Covariates > Clinical Covariates	0.0136	2.2098
Cam_121 > Clinical Covariates	0.0202	2.0502

Supplementary Table S2c: Statistical analysis of mean classifier results shown in Figure 3e (bootstrap). The mean performances of 7 classifiers for signatures were compared in a pairwise manner using one-sided two-sample Welch t-tests implemented in R by the function t.test() with parameters (var.equal = FALSE, paired = FALSE, alternative = "greater"). No multiplicity correction were used.

	ROC		Sensitivity		Specificity	
Signature1 > Signature2	p-value	z-statistic	p-value	z-statistic	p-value	z-statistic
Cam_121 + Clinical Covariates > Clinical Covariates	0.000387	4.07E+00	3.47E-04	3.8534	0.6684	-0.4411
Cam_121 > Clinical Covariates	0.000703	4.4371	0.0283	2.1533	0.3698	0.3419
LMC_150 > Clinical Covariates	0.109007	1.3209	0.2835	0.5928	0.2565	0.6755
Gerami_27 > Clinical covariates	0.105969	1.3439	0.1579	1.0642	0.4710	0.0749
Cam_121 > LMC_150	0.015221	2.4532	0.1208	1.2322	0.5786	-0.2030
Cam_121 > Gerami_27	0.020351	2.2962	0.2679	0.6385	0.4215	0.2023

Supplementary Table S2d: Statistical analysis of mean classifier results shown in Figure 3e (10-Fold cross validation). The mean performances of 7 classifiers for signatures were compared in a pairwise manner using one-sided Welch Two Sample t-tests implemented in R by the function t.test() with parameters (var.equal = FALSE, paired = FALSE, alternative = "greater")

	ROC		Sensitivity		Specificity	
Signature1 > Signature2	p-value	z-statistic	p-value	z-statistic	p-value	z-statistic
Cam_121 + Clinical Covariates > Clinical Covariates	3.87E-04	4.07E+00	3.47E-04	3.85E+00	6.68E-01	-4.41E-01
Cam_121 > Clinical Covariates	5.43E-02	1.83E+00	1.34E-02	2.53E+00	8.08E-01	-9.22E-01
LMC_150 > Clinical Covariates	4.86E-01	3.73E-02	9.52E-02	1.39E+00	8.06E-01	-9.16E-01
Gerami_27 Melanoma > Clinical covariates	1.71E-01	9.95E-01	1.07E-01	1.31E+00	7.28E-01	-6.26E-01
Cam_121 > LMC_150	8.71E-02	1.45E+00	2.55E-01	6.83E-01	4.88E-01	3.13E-02
Cam_121 > Gerami_27 Melanoma	1.35E-01	1.18E+00	1.38E-01	1.14E+00	6.93E-01	-5.22E-01

Supplementary Table S3: Distribution of clinical covariates across the high versus low risk signature groups. Please note that the corresponding two-sided statistical test used to obtain the p-values is indicated in the last column of the table. No multiplicity correction were used.

Variable	Levels	Statistics	Overall	Signature - high	Signature - low	P-value	Statistical test
Full dataset		n	194	130	64		
	Male	n (%)	123 (63.4%)	83 (63.8%)	40 (62.5%)		
	Female	n (%)	71 (36.6%)	47 (36.2%)	24 (37.5%)		
Sex	Missing	n (%)	0 (0.0%)	0 (0.0%)	0 (0.0%)	0.9804	Chi-square test
Age		Median (IQR)	57.0 (44.0-66.0)	57.5 (44.0-66.0)	56.0 (43.5-65.2)	0.9375	Welch's test
	Head and neck	n (%)	39 (20.1%)	25 (19.2%)	14 (21.9%)		
	Lower limbs	n (%)	55 (28.4%)	41 (31.5%)	14 (21.9%)		
	Trunk	n (%)	71 (36.6%)	47 (36.2%)	24 (37.5%)		
	Upper limbs	n (%)	29 (14.9%)	17 (13.1%)	12 (18.8%)		
Site	Missing	n (%)	0 (0.0%)	0 (0.0%)	0 (0.0%)	0.4741	Chi-square test
	<= 2.0 mm	n (%)	31 (16.0%)	22 (16.9%)	9 (14.1%)		
	>2-4mm	n (%)	63 (32.5%)	40 (30.8%)	23 (35.9%)		
	>4.0mm	n (%)	100 (51.5%)	68 (52.3%)	32 (50.0%)		
Bres	Missing	n (%)	0 (0.0%)	0 (0.0%)	0 (0.0%)	0.7347	Chi-square test
	Present	n (%)	104 (53.6%)	66 (50.8%)	38 (59.4%)		
	Absent	n (%)	83 (42.8%)	57 (43.8%)	26 (40.6%)		
Ulc	Missing	n (%)	7 (3.6%)	7 (5.4%)	0 (0.0%)	0.5543	Chi-square test
	N0	n (%)	80 (41.2%)	44 (33.8%)	36 (56.2%)		
	N1a	n (%)	32 (16.5%)	25 (19.2%)	7 (10.9%)		
	N1b	n (%)	17 (8.8%)	11 (8.5%)	6 (9.4%)		
	N2a	n (%)	19 (9.8%)	17 (13.1%)	2 (3.1%)		
	N2b	n (%)	5 (2.6%)	3 (2.3%)	2 (3.1%)		
Nclass	N2c	n (%)	13 (6.7%)	7 (5.4%)	6 (9.4%)	0.0115	Fisher's test

Supplementary Table S3: Distribution of clinical covariates across the high versus low risk signature groups (continued)

Variable	Levels	Statistics	Overall	Signature - high	Signature - low	P-value	Statistical test
	N3	n (%)	18 (9.3%)	16 (12.3%)	2 (3.1%)		
	Missing	n (%)	10 (5.2%)	7 (5.4%)	3 (4.7%)		
Nclass_binary	Positive	n (%)	104 (53.6%)	79 (60.8%)	25 (39.1%)	0.0046	Chi-square test
	Negative	n (%)	80 (41.2%)	44 (33.8%)	36 (56.2%)		
	Missing	n (%)	10 (5.2%)	7 (5.4%)	3 (4.7%)		
Stage	IIB	n (%)	45 (23.2%)	21 (16.2%)	24 (37.5%)	0.0145	Fisher's test
	IIC	n (%)	45 (23.2%)	30 (23.1%)	15 (23.4%)		
	IIIA	n (%)	29 (14.9%)	23 (17.7%)	6 (9.4%)		
	IIIB	n (%)	51 (26.3%)	37 (28.5%)	14 (21.9%)		
	IIIC	n (%)	24 (12.4%)	19 (14.6%)	5 (7.8%)		
	Missing	n (%)	0 (0.0%)	0 (0.0%)	0 (0.0%)		
Stage_binary	II	n (%)	90 (46.4%)	51 (39.2%)	39 (60.9%)	0.007	Chi-square test
	III	n (%)	104 (53.6%)	79 (60.8%)	25 (39.1%)		
	Missing	n (%)	0 (0.0%)	0 (0.0%)	0 (0.0%)		
BRAF	V600E	n (%)	75 (38.7%)	50 (38.5%)	25 (39.1%)	1	Chi-square test
	WT	n (%)	104 (53.6%)	69 (53.1%)	35 (54.7%)		
	Missing	n (%)	15 (7.7%)	11 (8.5%)	4 (6.2%)		
NRAS	Mutant	n (%)	35 (18.0%)	23 (17.7%)	12 (18.8%)	1	Chi-square test
	WT	n (%)	63 (32.5%)	40 (30.8%)	23 (35.9%)		
	Missing	n (%)	96 (49.5%)	67 (51.5%)	29 (45.3%)		
BRAF_NRAS_WT	Yes	n (%)	70 (36.1%)	47 (36.2%)	23 (35.9%)	1	Chi-square test
	No	n (%)	109 (56.2%)	72 (55.4%)	37 (57.8%)		
	Missing	n (%)	15 (7.7%)	11 (8.5%)	4 (6.2%)		

Supplementary Table S3: Distribution of clinical covariates across the high versus low risk signature groups (continued)

Variable	Levels	Statistics	Overall	Signature - high	Signature - low	P-value	Statistical test
	Bevacizumab	n (%)	100 (51.5%)	68 (52.3%)	32 (50.0%)		
	Observation	n (%)	94 (48.5%)	62 (47.7%)	32 (50.0%)		
	Missing	n (%)	0 (0.0%)	0 (0.0%)	0 (0.0%)		
treatment	Missing	n (%)	0 (0.0%)	0 (0.0%)	0 (0.0%)	0.8811	Chi-square test
	Symptomatic	n (%)	16 (8.2%)	13 (10.0%)	3 (4.7%)		
	Asymptomatic	n (%)	178 (91.8%)	117 (90.0%)	61 (95.3%)		
ECOG	Missing	n (%)	0 (0.0%)	0 (0.0%)	0 (0.0%)	0.2725	Fisher's test
	Yes	n (%)	89 (45.9%)	71 (54.6%)	18 (28.1%)		
	No	n (%)	105 (54.1%)	59 (45.4%)	46 (71.9%)		
EventMet	Missing	n (%)	0 (0.0%)	0 (0.0%)	0 (0.0%)	0.0009	Chi-square test
