# Supplementary material for: Tumour gene expression signature in primary melanoma predicts long-term outcomes
Source: Nat Commun. 2021 Feb 18;12:1137. doi: 10.1038/s41467-021-21207-2 (PMC7893180; doi:10.1038/s41467-021-21207-2)
Supplement: Supplementary file 3 — Description of Additional Supplementary Files [file 41467_2021_21207_MOESM3_ESM.docx]

**Description of Additional Supplementary Files**

File Name: Supplementary Data 1

Description: The description of genes in the Cam_121 signature. Gene list is sorted in decreasing order based on the shrunken log2(fold-change) values (see Methods section 9). The Wald test p-values were adjusted for multiple comparisons using Benjamini-Hochberg Procedure and are reported in the column "padj". The corresponding beta-coefficient values used to calculate the weighted gene expression scores in the current paper (equation 1) are given in the column "BetaCoeff".

File Name: Supplementary Data 2

Description: Survival analysis per gene across all protein-coding genes in multivariate Cox regression model (overall survival). The Wald t-test p-values corresponding to each gene are indicated for each model and outcome under the column “p-value”. HR: Hazard Ratio; CI: confidence interval; padj.BH: p-values after multiplicity correction using Benjamini Hochberg procedure; padj.Fdr: false discovery rate corrected p-values.

File Name: Supplementary Data 3

Description: Survival analysis per gene across all protein-coding genes in multivariate Cox regression model (progression-free survival). The Wald t-test p-values corresponding to each gene are indicated for each model and outcome under the column “p-value”. HR: Hazard Ratio; CI: confidence interval; padj.BH: p-values after multiplicity correction using Benjamini Hochberg procedure; padj.Fdr: false discovery rate corrected p-values.
