# Supplementary material for: Tumour gene expression signature in primary melanoma predicts long-term outcomes
Source: Nat Commun. 2021 Feb 18;12:1137. doi: 10.1038/s41467-021-21207-2 (PMC7893180; doi:10.1038/s41467-021-21207-2)
Supplement: Supplementary file 7 — Reporting Summary [file 41467_2021_21207_MOESM7_ESM.pdf]

nature research

Corresponding author(s): Dr Roy Rabbie

Last updated by author(s): Jan 25, 2021

Reporting Summary

Nature Research wishes to improve the reproducibility of the work that we publish. This form provides structure for consistency and transparency in reporting. For further information on Nature Research policies, see our Editorial Policies and the Editorial Policy Checklist.

Statistics

For all statistical analyses, confirm that the following items are present in the figure legend, table legend, main text, or Methods section.

n/a	Confirmed	
☐	☒	The exact sample size (*n*) for each experimental group/condition, given as a discrete number and unit of measurement
☐	☒	A statement on whether measurements were taken from distinct samples or whether the same sample was measured repeatedly
☐	☒	The statistical test(s) used AND whether they are one- or two-sided<br>*Only common tests should be described solely by name; describe more complex techniques in the Methods section.*
☐	☒	A description of all covariates tested
☐	☒	A description of any assumptions or corrections, such as tests of normality and adjustment for multiple comparisons
☐	☒	A full description of the statistical parameters including central tendency (e.g. means) or other basic estimates (e.g. regression coefficient) AND variation (e.g. standard deviation) or associated estimates of uncertainty (e.g. confidence intervals)
☐	☒	For null hypothesis testing, the test statistic (e.g. *F*, *t*, *r*) with confidence intervals, effect sizes, degrees of freedom and *P* value noted<br>*Give P values as exact values whenever suitable.*
☒	☐	For Bayesian analysis, information on the choice of priors and Markov chain Monte Carlo settings
☐	☒	For hierarchical and complex designs, identification of the appropriate level for tests and full reporting of outcomes
☒	☐	Estimates of effect sizes (e.g. Cohen's *d*, Pearson's *r*), indicating how they were calculated

Our web collection on statistics for biologists contains articles on many of the points above.

Software and code

Policy information about availability of computer code

Data collection	No software was used in the data collection for this study.

Data analysis

The full code for the analyses within this study is available on GitHub (https://github.com/Manikgarg/MelanomaTranscriptomics/tree/master/scripts). The R-packages used for data analysis are indicated within the manuscript and listed below:
coin (v1.3-1)
DESeq2 (v1.18)
apeglm (v1.6.0)
survival (v3.2.3)
caret (v6.0-86)
snakemake (v5.17.0)
pROC (v1.16.2)
VennDiagram (v1.6.20)
ggplot2 (v 3.3.0)
ggpubr (v0.2.5)
graphics (v3.6.3)
stats (v 3.6.2)
SigCheck (v2.14.0)
survminer (v0.4.7)

In addition to these R-packages, we used fastq_utils (v0.14.7) and FastQC (v0.11.7) for quality control (QC) of mRNA reads, TopHat2 for QC passed mRNA reads alignment and HTSeq for aligned reads quantification. The GSEA software (v4.0.2) from Broad Institute was used to perform gene set enrichment analyses.

For manuscripts utilizing custom algorithms or software that are central to the research but not yet described in published literature, software must be made available to editors and reviewers. We strongly encourage code deposition in a community repository (e.g. GitHub). See the Nature Research guidelines for submitting code & software for further information.

Data

Policy information about availability of data

All manuscripts must include a data availability statement. This statement should provide the following information, where applicable:

- Accession codes, unique identifiers, or web links for publicly available datasets
- A list of figures that have associated raw data
- A description of any restrictions on data availability

All source data are provided with this paper. The raw RNA sequencing data (forward and reverse fastq files) has been made available at the European Genome-Phenome Archive at the EBI under the following dataset accession ID: EGAD00001006401 [https://www.ebi.ac.uk/ega/datasets/EGAD00001006401]. The source data underlying Figures 3, 4 and 5; Supplementary Figures S2, S3b, S7, S8, S10, S13 and S14 (b and c); Table 1 and Supplementary Table S2 are provided as a Source Data file within this paper, as well as through the GitHub repository: Manikgarg/MelanomaTranscriptomics [https://github.com/Manikgarg/MelanomaTranscriptomics/tree/master/Source_Data] (64). The clinical and gene expression data from The Cancer Genome Atlas (TCGA-SKCM), can be downloaded from the cBioPortal (37). Data from the Leeds Melanoma Cohort (16), Lund Melanoma Cohort (22) and the Australia Melanoma Genome Project (23) are available from the source publications. The MSigDB database (60) gene set collections are available for download from http://www.gsea-msigdb.org/gsea/downloads.jsp#msigdb

Field-specific reporting

Please select the one below that is the best fit for your research. If you are not sure, read the appropriate sections before making your selection.

☒ Life sciences ☐ Behavioural & social sciences ☐ Ecological, evolutionary & environmental sciences

For a reference copy of the document with all sections, see nature.com/documents/nr-reporting-summary-flat.pdf

Life sciences study design

All studies must disclose on these points even when the disclosure is negative.

Sample size

All samples with RNA sequencing data available (N=446) from the source publication; Corrie et al. Lancet Oncol 2014 (PMID: 24745696), were included within this analysis. A power calculation was undertaken to determine the minimum sample size required for the external validation of our findings (see methods section 16 and Supplementary Figure S4b).

Data exclusions

Non-primary melanoma samples (including those labeled as; lymph node (n=177), local/distant relapse (n=58) and uncategorised (n=7)) were excluded from the primary analysis. Similarly, primary melanoma samples with missing data relating to the corrected clinical covariates (Stage, Breslow thickness, ECOG PS, treatment) were excluded (n=10). A summary flowchart of the excluded samples and downstream analyses is outlined in Supplementary Figure S1.

Replication

Internal replication of the Cox regression survival analyses was undertaken using the regional lymph node samples (N=143).
External replication of the Cox regression survival analyses was undertaken using data from The Cancer Genome Atlas (TCGA-SKCM) (21), Leeds Melanoma Cohort (16), Lund Melanoma Cohort (22) and the Australia Melanoma Genome Project (23).

Internal replication of the machine learning analyses was undertaken using the regional lymph node samples (N=143).
External replication of the machine learning analyses was not possible owing to a lack of suitable datasets.

A summary flowchart of the analyses and replication datasets is outlined in Supplementary Figure S1.

Randomization	Risk-scoring of samples (according to the Cam_121 gene signature) was undertaken using covariate-corrected differential expression analysis between patients with distant relapse vs those with no distant relapse. Full details are available within the Methods section 9.
Blinding	Blinding was not possible in this study. The clinical outcomes were determined using well established clinical criteria, as defined within the Methods section 12.

Reporting for specific materials, systems and methods

We require information from authors about some types of materials, experimental systems and methods used in many studies. Here, indicate whether each material, system or method listed is relevant to your study. If you are not sure if a list item applies to your research, read the appropriate section before selecting a response.

Materials & experimental systems

n/a	Involved in the study	
☒	☐	Antibodies
☒	☐	Eukaryotic cell lines
☒	☐	Palaeontology and archaeology
☒	☐	Animals and other organisms
☐	☒	Human research participants
☐	☒	Clinical data
☒	☐	Dual use research of concern

Methods

n/a	Involved in the study	
☒	☐	ChIP-seq
☒	☐	Flow cytometry
☒	☐	MRI-based neuroimaging

Human research participants

Policy information about studies involving human research participants

Population characteristics	Eligible patients were at least 16 years old, with histological confirmation of completely resected American Joint Committee 7th edition stage IIB (T3bN0M0 and T4aN0M0), IIC (T4bN0M0), or III (TxN1–3M0) cutaneous melanoma. Inclusion criteria also included an Eastern Cooperative Oncology Group (ECOG) performance status of 0–1; a life expectancy of 6 months or more; and adequate haematological, liver, and renal function. See full trial protocol for further full details, PMID: 24745696.
Recruitment	Patients were recruited at one of the 48 participating centres in the UK between July 18, 2007, and March 29, 2012.
Ethics oversight	The trial protocol (including the collection of DNA and RNA) was ethically approved by the National Research Ethics Committee in accordance with the Declaration of Helsinki (REC reference number 07/Q1606/15, 16th March 2007).

Note that full information on the approval of the study protocol must also be provided in the manuscript.

Clinical data

Policy information about clinical studies

All manuscripts should comply with the ICMJE guidelines for publication of clinical research and a completed CONSORT checklist must be included with all submissions.

Clinical trial registration	This trial is registered as an International Standardised Randomised Controlled Trial, number ISRCTN81261306
Study protocol	Available through Corrie, P.G. et al. Adjuvant bevacizumab in patients with melanoma at high risk of recurrence (AVAST-M): preplanned interim results from a multicentre, open-label, randomised controlled phase 3 study. Lancet Oncol 15, 620-30 (2014), PMID: 24745696.
Data collection	Available through Corrie, P.G. et al. Adjuvant bevacizumab in patients with melanoma at high risk of recurrence (AVAST-M): preplanned interim results from a multicentre, open-label, randomised controlled phase 3 study. Lancet Oncol 15, 620-30 (2014), PMID: 24745696.
Outcomes	Available through Corrie, P.G. et al. Adjuvant bevacizumab in patients with melanoma at high risk of recurrence (AVAST-M): preplanned interim results from a multicentre, open-label, randomised controlled phase 3 study. Lancet Oncol 15, 620-30 (2014), PMID: 24745696.
